# Suppression of the invasive potential of Glioblastoma cells by mTOR inhibitors involves modulation of NFκB and PKC-α signaling

**DOI:** 10.1038/srep22455

**Published:** 2016-03-04

**Authors:** Goparaju Chandrika, Kumar Natesh, Deepak Ranade, Ashish Chugh, Padma Shastry

**Affiliations:** 1 National Centre for Cell Science (NCCS), Savitribai Phule Pune University Campus, Pune, India; 2 Department of Neurosurgery, D.Y. Patil Medical College, Pune, India; 3 Department of Neurosurgery, Cimet’s Inamdar Multispecialty Hospital, Pune, India

## Abstract

Glioblastoma (GBM) is the most aggressive type of brain tumors in adults with survival period <1.5 years of patients. The role of mTOR pathway is documented in invasion and migration, the features associated with aggressive phenotype in human GBM. However, most of the preclinical and clinical studies with mTOR inhibitors are focused on antiproliferative and cytotoxic activity in GBM. In this study, we demonstrate that mTOR inhibitors-rapamycin (RAP), temisirolimus (TEM), torin-1 (TOR) and PP242 suppress invasion and migration induced by Tumor Necrosis Factor-α (TNFα) and tumor promoter, Phorbol 12-myristate 13-acetate (PMA) and also reduce the expression of the TNFα and IL1β suggesting their potential to regulate factors in microenvironment that support tumor progression. The mTOR inhibitors significantly decreased MMP-2 and MMP-9 mRNA, protein and activity that was enhanced by TNFα and PMA. The effect was mediated through reduction of Protein kinase C alpha (PKC-α) activity and downregulation of NFκB. TNFα- induced transcripts of NFκB targets -VEGF, pentraxin-3, cathepsin-B and paxillin, crucial in invasion were restored to basal level by these inhibitors. With limited therapeutic interventions currently available for GBM, our findings are significant and suggest that mTOR inhibitors may be explored as anti-invasive drugs for GBM treatment.

Glioblastoma (GBM) is the highly predominant form of life threatening primary malignant gliomas and astrocytomas. It is primarily characterized by genetic instability, intra-tumoral histopathological variability and unpredictable patient survival probability^1^,^2^. The clinical hallmarks of GBM include aggressive proliferation and persistent recurrence due to invasive infiltration into the surrounding brain tissue despite multimodal therapy that comprises surgery accompanied by radiation and chemotherapy^3^,^4^. GBM (Grade IV astrocytoma) shows extremely poor prognosis with survival period of less than 1.5 years in patients. Conventional therapy for GBM is treatment with temozolomide (TMZ) in combination with radiation therapy^5^,^6^. However, in most cases, this is followed by intrinsic or acquired resistance to TMZ resulting in complications and failure of treatment^7^,^8^.

Extensive aberrations of gene expression profiles found among GBMs greatly affect cellular invasion potential, angiogenesis, immune cell infiltration, and extracellular matrix remodelling related to cell migration. Occurrence of highly deregulated tumor genome with opportunistic deletion of tumor suppressor genes, amplification and/or mutational hyper-activation of Receptor Tyrosine Kinase receptors result in augmented survival, proliferation and invasion pathways^9^,^10^.

The mammalian Target of Rapamycin (mTOR) signaling network downstream in EGFR/PI3K/Akt pathway regulates cell growth, proliferation, and survival^11^. The central component of the pathway, the mTOR protein kinase, nucleates two distinct multi-protein complexes that regulate different branches of the mTOR network. The mTOR complex 1 (mTORC1) consists of mTOR, raptor and mLST8. It regulates cell growth translational machinery through effectors such as Ribosomal protein S6 kinase beta-1 (S6K1) and eukaryotic initiation factor 4E-binding protein 1 (4EBP1). The mTOR complex 2 (mTORC2) contains mTOR, rictor, Sin-1 and mLST8 and modulates the actin cytoskeletal functioning (RhoA, Rac1) through Protein kinase C alpha (PKC-α) and pro-survival Protein kinase B (Akt/PKB) by phosphorylating it on S473^12^.

The mTOR pathway is highly activated in GBMs and one of the most studied inhibitors of mTOR is Rapamycin (RAP), an FDA approved drug that works through a gain-of-function allosteric mechanism. RAP binds to the intracellular protein FKBP12 to generate a drug-receptor complex that binds to and inhibits the kinase activity of mTORC1^13^. Subsequent reports demonstrated that prolonged treatment with RAP in various cell types suppressed the assembly and function of mTORC2 to inhibit Akt/PKB^14^. Rapamycin and its analogs have been used in combination with radiation, PI3K and ERK inhibitors to demonstrate its effectiveness to treat GBM patients^15^. An improved version of RAP, Temisirolimus (TEM), a water-soluble ester derivative of RAP is approved by FDA. Since TEM crosses Blood Brain Barrier, it is presently under phase II clinical trials individually as well as in combination with other drugs to treat GBM^16^,^17^. The general anticancer activity shown by original mTOR allosteric inhibitors, RAP and its analogs (rapalogs) in most cancers, has supported the development of novel mTOR kinase inhibitors (TORKinibs) that inhibit mTORC1 and mTORC2 more effectively^18^. TORKinibs such as Torin-1 (TOR) and PP-242 are potent and selective small molecule inhibitors that bind to ATP binding site of mTOR molecule and efficiently inhibit, mTORC1 as well as mTORC2 complexes. The mechanism of action of TORKinibs is different from that of rapalogs as they can prevent cap dependent translational process^19^,^20^.

Invasiveness of GBM tumors is one of the characteristic hallmarks that contributes to tumor recurrence. Therefore in-depth studies aiming to further understand this process are crucial to develop improved therapies^21^,^22^. Targeted inhibition of mTOR pathway has been studied extensively to control tumor growth and sustenance but not sufficiently understood to explore its implications to control tumor invasion and recurrence. In this study, we investigated the anti-invasive and -migration potential of mTOR inhibitors (RAP, TEM, TOR and PP242) in human GBM cells. We show that the mTOR inhibitors suppressed invasion and migration in GBM cells in the presence of TNFα and tumor promoter PMA mediated by reduction of PKC-α activity and downregulation of NFκB.

## Results

### Effect of mTOR inhibitors on cell viability and mTOR signaling in GBM cells

**Figure 1:**
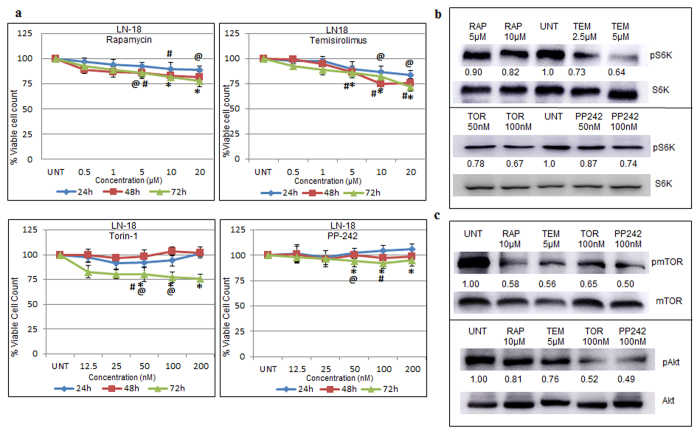
Effect of mTOR inhibitors on cell viability and mTOR signaling in GBM cells. (**a**) LN-18 cells were treated with serial concentrations of rapamycin-RAP, temisirolimus-TEM, torin-TOR and PP-242 for 24 h, 48 h and 72 h and percentage of viable cell count was assessed by MTT assay. Viable count of untreated cells was assumed as 100%. The graphs represent % viable cell count +/− SEM of three similar experiments performed in triplicates. ^@^p-value < 0.05 Untreated vs. inhibitor treatment for 24 h; ^#^p-value < 0.05 Untreated vs. inhibitor treatment for 48 h; *p–value < 0.05 Untreated vs. inhibitor treatment for 72 h. (**b**) Protein levels of phospho S6K (Thr 389) and total S6K were measured by immunoblotting of total cell lysates of LN-18 cells treated with two concentrations of RAP (5 μM, 10 μM), TEM (2.5 μM, 5 μM), TOR (50 nM, 100 nM) or PP-242 (50 nM, 100 nM) for 24 h. Representative cropped images of two independent experiments. (**c**) Protein levels of phospho Akt (Ser 473), total Akt, phospho mTOR (Ser2448) and total mTOR were measured by immunoblotting of total cell lysates of LN-18 cells treated with RAP (10 μM), TEM (5 μM), TOR (100 nM) or PP-242 (100 nM) for 24 h. Representative cropped images of three independent experiments. Images show fold change of treated phospho-S6K or -Akt or -mTOR protein expression (normalised with respect to total-S6K or -Akt or -mTOR) relative to untreated samples obtained by densitometry through ImageJ analysis. Full length blots are included in supplementary Fig. S10.

Dose and time dependent effect of mTOR inhibitors on cell survival and proliferation was assessed in LN-18 cell line and primary cultures G-1 by MTT assay. In LN-18 cells, the viable cell count was reduced by ~20–25% on treatment for 72 h with mTOR inhibitors except for PP242 (Fig. 1a). In G-1 cells, the mTOR inhibitors except for RAP reduced the viable cell count by ~20–25% at 72 h of treatment (Supplementary Fig. S1). To examine if the effect of inhibitors was sustained, “washout” experiments were performed. Cells were treated with inhibitors for 24 h or 48 h followed by change of medium using fresh medium without inhibitors and incubated further for 24 h and 48 h respectively. As depicted in Supplementary Fig. S2, the viable cell count in “washout” samples were comparable to samples treated with inhibitors for 72 h, suggesting sustained antiproliferative activity of the mTOR inhibitors.

Previous path-breaking reports on mTOR pathway emphasized phospho p70-s6kinase and phospho Akt (Ser473) as standard readout proteins for kinase activity of mTORC1 and mTORC2 respectively^12^. In accordance, we found that the inhibitors - RAP, TEM, TOR and PP242 significantly depleted phosphorylated- mTOR, -S6K and -Akt (Ser473) in LN-18 cells confirming that the activity of both m-TOR complexes was effectively down regulated by the mTOR inhibitors (Fig. 1b,c). The inhibitors reduced the level of phospho Akt (Ser 473) more effectively than phospho S6K levels suggesting that the effect of the inhibitors on mTORC1 and mTORC2 may not be to the same extent in LN-18 cells.

A recent report suggested that mTOR-inhibitors differentially influence mitochondrial dynamics in cancer cells which might affect therapeutic efficiency of mTOR-targeted therapy^23^. To this end, we observed that treatment with RAP and TEM reduced the mitochondrial membrane potential (MMP) in GBM cells, but Torin and PP242 had no effect (Supplementary Fig. S3).

### Inhibition of the invasive potential by mTOR inhibitors

**Figure 2:**
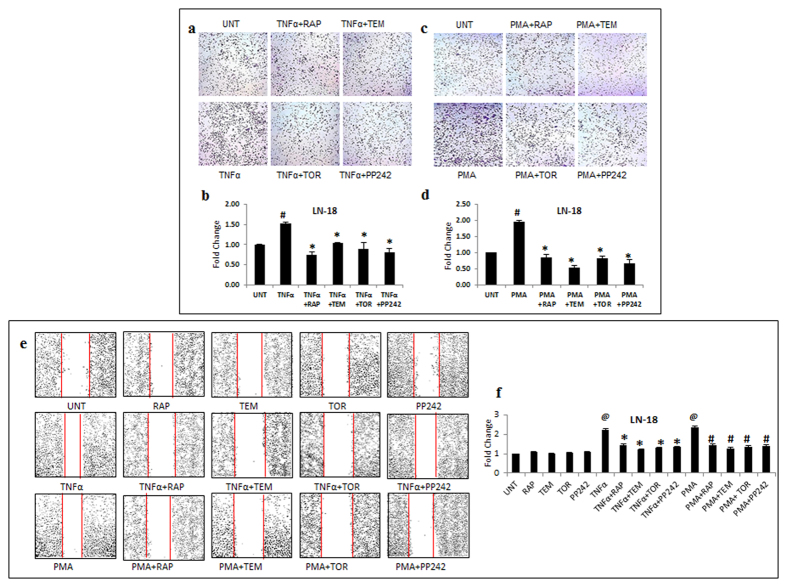
mTOR inhibitors inhibit invasion and migration potential in GBM. LN-18 cells treated with TNFα (10 ng/ml) or PMA (100 ng/ml) alone or in combination with inhibitors- rapamycin-RAP (10 μM), temisirolimus-TEM (5 μM), torin-TOR (100 nM) or PP-242 (100 nM) for 24 h were used to perform matrigel invasion assay and scratch wound healing assay. Representative images of invasion in LN-18 cells treated with (**a**) TNFα and (**c**) PMA. (**b**) and (**d**) The graphs represent fold change in invasive cell number +/− SEM of two similar experiments performed in duplicates. ^#^p-value < 0.05 Untreated vs. TNFα or PMA treatment; *p–value < 0.05 TNFα or PMA treatment alone vs. TNFα or PMA in combination with inhibitor treatment. (**e**) Representative images of wound closure in LN-18 cells exposed to TNFα or PMA alone or in combination with inhibitors. (**f**) The graph represents fold change in width of wound closure +/− SEM of three similar experiments performed in duplicates. ^@^p-value < 0.05 Untreated vs. TNFα or PMA treatment; *p–value < 0.05 TNFα treatment vs. TNFα in combination with inhibitors; ^#^p–value < 0.05 PMA treatment vs. PMA in combination with inhibitors.

**Figure 3:**
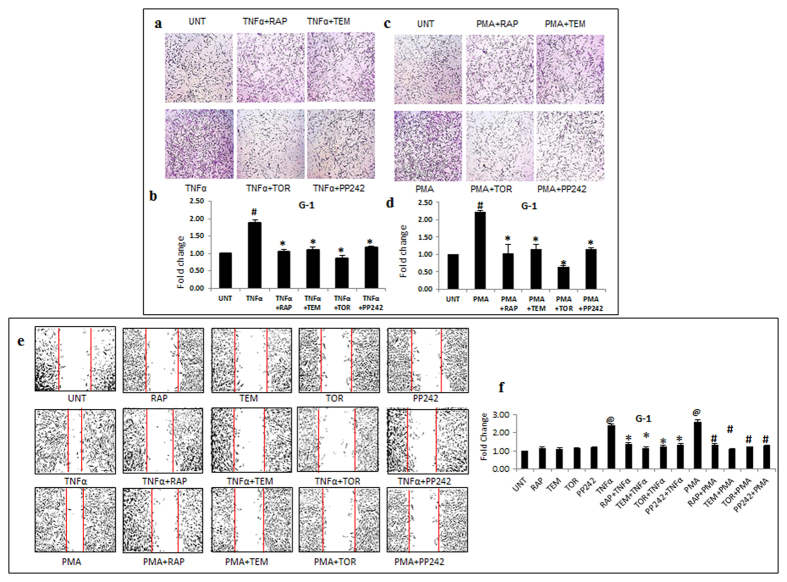
mTOR inhibitors inhibit invasion and migration potential in GBM primary cell culture. G-1 cells treated with TNFα (10 ng/ml) or PMA (100 ng/ml) alone or in combination with inhibitors- rapamycin-RAP (10 μM), temisirolimus-TEM (5 μM), torin-TOR (100 nM) or PP-242 (100 nM) for 24 h were used to perform matrigel invasion assay and scratch wound healing assay. Representative images of invasion in G-1 cells treated with (**a**) TNFα and (**c**) PMA. (**b**,**d**) The graphs represent fold change in invasive cell number +/− SEM of two similar experiments performed in duplicates. ^#^p-value < 0.05 Untreated vs. TNFα or PMA treatment; *p-value < 0.05 TNFα or PMA treatment vs. TNFα or PMA in combination with inhibitors. (**e**) Representative images of wound closure in primary culture G-1 cells exposed to TNFα or PMA alone or in combination with inhibitors. (**f**) The graph represents fold change in width of wound closure +/− SEM of three similar experiments performed in duplicates. ^@^p-value < 0.05 Untreated vs. TNFα or PMA treatment; *p-value < 0.05 TNFα treatment vs. TNFα in combination with inhibitors; ^#^p-value < 0.05 PMA treatment vs. PMA in combination with inhibitors.

We further evaluated the influence of mTOR inhibitors on more aggressive traits that cause recurrence and invasion into surrounding tissues. Considering that TNFα and PMA trigger signaling pathways that are crucial in inducing enhanced aggressiveness, we exposed LN-18 and G1 cells to TNFα or PMA followed by treatment with inhibitors. Cellular invasion, an important function was assessed by matrigel matrix invasion assay. In LN-18 cells, TNFα and PMA enhanced the invasive potential to 1.5 and 2 fold respectively (p ≤ 0.05). All the four mTOR inhibitors significantly reduced the invasion induced by TNFα and PMA (Fig. 2a–d). The effect of TNFα and PMA was more robust in primary culture (G-1) with the increase in invasive potential up to 2 and 2.25 fold (p ≤ 0.05) respectively and treatment with the inhibitors restored the basal level (Fig. 3a–d). Another feature of resilient tumors- cell migration was monitored by scratch wound healing assay. TNFα and PMA enhanced cell migration in LN-18 cells to 2.25 and 2.4 fold (p ≤ 0.05) respectively (Fig. 2e,f) and in G-1 cells to 2.4 and 2.6 fold (p ≤ 0.05) respectively (Fig. 3e,f). In LN-18 and G-1 cells, all the mTOR inhibitors limited the migration induced by TNFα and PMA. There was no significant difference in the effectiveness between the mTOR inhibitors. Collectively, these results confirmed that mTOR inhibitors can restrain diverse cellular responses related to aggressiveness in GBM cells.

### mTOR inhibitors reduce the induced- gelatinolytic MMPs

**Figure 4:**
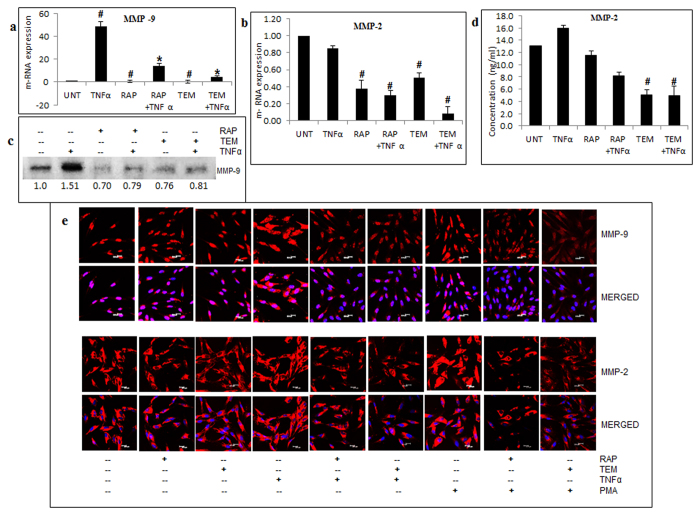
mTOR inhibitors regress induced-gelatinolytic MMPs. LN-18 cells were treated with rapamycin-RAP (10 μM) or temisirolimus-TEM (5 μM) for 24 h alone and in combination with TNFα (10 ng/ml) for 12 h and transcript level of (**a**) MMP-9 and (**b**) MMP-2 was measured by real-time PCR. GAPDH was used as constitutionally active internal control. The graph represents fold change in relative m-RNA expression +/− SEM of two similar experiments performed in triplicates. ^#^p-value < 0.05 Untreated vs. TNFα or inhibitor treated; *p–value < 0.05 TNFα treatment vs. TNFα in combination with inhibitor treatment. Conditioned media of LN-18 cells treated with RAP (10 μM) or TEM (5 μM) for 24 h alone and in combination with TNFα (10 ng/ml) for 12 h before termination of time point were used to measure protein expression of MMP-9 by immunoblotting and MMP-2 by ELISA. (**c**) Representative cropped image showing fold change of MMP-9 protein expression relative to untreated samples obtained by densitometry through ImageJ analysis. Full length blot is included in supplementary Fig. S11. (**d**) The graph represents values of protein concentrations (Y-axis) +/− SEM of two similar experiments performed in triplicates. ^#^p-value < 0.05 Untreated vs. TNFα or inhibitor or combination treatment. (**e**) Immunofluorescence staining for MMP-9 and MMP-2 protein intensity performed on G-1 cells treated using RAP (10 μM) or TEM (5 μM) alone and in combination with TNFα (10 ng/ml) for 12 h before termination of time point or PMA (100 ng/ml) for 24 h. Nuclear staining with DAPI. Representative images of three independent experiments. Scale: 20 μm.

The matrix metalloproteinases (MMPs) play a key role in tumor cell invasion, metastasis and angiogenesis by promoting ECM degradation and processing of cytokines, growth factors, hormones and cell receptors^24^,^25^. Further experiments were performed to examine the effect of mTOR inhibitors on the expression and activity of MMP-2 and MMP-9 that are highly expressed in GBM^26^. Consistent with earlier reports^27^, Real time PCR analysis revealed that TNFα drastically elevated MMP-9 mRNA to 50 ± 4 fold (p ≤ 0.05) in LN-18 cells. This finding is not surprising as MMP-9 is a target of NFκB and TNFα is a classical activator of this signaling pathway. This induced MMP-9 was effectively reduced by 35 ± 2 fold by RAP and 45 ± 1 fold by TEM (p ≤ 0.05) (Fig. 4a). Western blot analysis of supernatants revealed that TNFα- induced MMP-9 protein level (1.5 fold) was decreased by 0.7 fold by RAP and 0.6 fold by TEM (Fig. 4c). Constitutively expressed MMP-2 mRNA was reduced by 0.6 and 0.5 fold by RAP and TEM respectively (Fig. 4b). Estimation of MMP-2 protein which is regulated by PKC-α^28^ was performed by ELISA. Results indicated that reduction in the level of MMP-2 by TEM was more effective compared to RAP in the absence and presence of TNFα (Fig. 4d). Additionally, immunofluorescence experiments revealed that RAP and TEM effectively restored basal level of MMP-9 and MMP-2 induced by TNFα and PMA in G-1 cells (Fig. 4e).

**Figure 5:**
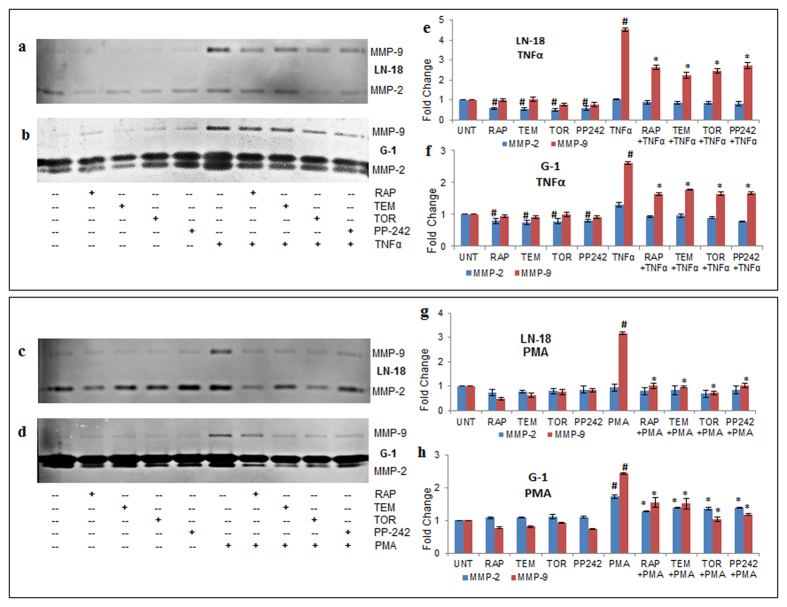
mTOR inhibitors revert induced-gelatinolytic MMP activity. Gelatinases-MMP-9 (92 KDa) and MMP-2 (72 KDa) functional activity was assessed by gelatin zymography in conditioned media of cells treated using rapamycin-RAP (10 μM), temisirolimus-TEM (5 μM), torin-TOR (100 nM), PP-242 (100 nM) alone and in combination with TNFα (10 ng/ml) for 12 h before termination of time point or PMA (100 ng/ml) for 24 h. Representative images of zymograms in (**a**) LN-18 and (**b**) G-1 cells treated by TNFα alone or in combination with inhibitors; (**c**) LN-18 and (**d**) G-1 cells treated by PMA alone or in combination with inhibitors. The graphs represent fold change in gelatinolytic enzyme activity +/− SEM of five independent experiments obtained by densitometry through ImageJ analysis for LN-18 cells treated with -TNFα (**e**), -PMA (**g**) and for G-1 cells with -TNFα (**f**), -PMA (**h**). ^#^p-value < 0.05 Untreated vs. TNFα or PMA or inhibitor treated; *p-value < 0.05 TNFα or PMA treatment vs. TNFα or PMA in combination with inhibitors.

Matrix metalloproteinases (MMPs) are regulated at multiple stages such as transcriptional, translational, post-translational and most importantly at the functional activity^24^. The activity of secretory MMPs was measured in the supernatants of cells treated with inhibitors by gelatinolytic zymography. The results corroborated with mRNA and protein level data. In LN-18 cells, TNFα induced- MMP-9 activity of 4.5 fold was reduced to 2.6 (RAP), 2.2 (TEM), 2.4 (TOR), 2.7 (PP242) fold (p ≤ 0.05) while the constitutive MMP-2 activity was reduced by ~0.5 fold by the mTOR inhibitors (p ≤ 0.05) (Fig. 5a,e). In G-1 cells, TNFα- induced MMP-9 activity of 2.6 fold was reduced to ~1.6 fold by the mTOR inhibitors (p ≤ 0.05) while constitutive MMP-2 activity was reduced by ~0.25 fold by the mTOR inhibitors (p ≤ 0.05) (Fig. 5b,f). In LN-18 cells, PMA induced- MMP-9 (3.2 fold) was restored to ~1 fold by the mTOR inhibitors (p ≤ 0.05) while MMP-2 activity was not significantly affected in the presence of PMA (Fig. 5c,g). In G-1 cells, PMA induced- MMP-9 activity (2.4 fold) was reduced significantly (p ≤ 0.05) to 1.5 fold (RAP and TEM) and ~1 fold (TOR and PP242) and PMA induced- MMP-2 activity (1.75 fold) was reduced by ~0.3 fold by the mTOR inhibitors (p ≤ 0.05) (Fig. 5d,h). In G-1 cells, distinct pro-active and active bands of MMP-2^29^ were observed and both the bands were considerably reduced by the inhibitors.

IL1β, an inflammatory cytokine is an activator of NFκB pathway and is abundantly present in the tumor microenvironment of many solid tumors including gliomas^30^. It was therefore of interest to validate the impact of mTOR inhibitors on MMP-9 and MMP-2 activity stimulated by IL1β. Exposure to IL1β resulted in 4.8 and 3 fold increase of MMP-9 levels in LN-18 and G-1 cells that were reduced effectively by ~1.2 and ~1.5 fold respectively by the mTOR inhibitors (Supplementary Fig. S4a,b). Constitutively active MMP-2 and IL1β- induced MMP-2 were decreased by ~0.3 fold by the mTOR inhibitors in LN-18 cells. In G-1 cells, induced- MMP-2 activity (2 fold) was decreased by 50% by the mTOR inhibitors. Interestingly, in spite of heterogeneity between various primary cultures, experiments conducted with another primary culture GBM (G-16) ascertained that MMP-9 and MMP-2 activity levels were diminished by the mTOR inhibitors (Supplementary Fig. S5a–c), thus underscoring the effectiveness of the mTOR inhibitors for controlling invasion in GBM cells.

Tissue inhibitors of metalloproteases (TIMPs) regulate MMP activity at various levels. TIMP-1 inhibits MMP-9 by direct binding- physically at 1:1 ratio^25^.To examine whether the MMP-9 activity affected by the inhibitors was regulated by TIMP-1, m-RNA and protein levels were monitored. The data revealed that TIMP-1 remained unaltered in cells exposed to RAP and TEM in the presence or absence of TNFα (Supplementary Fig. S6a,b). Taken together, these findings strengthened the data that inhibiting mTOR pathway in GBM cells significantly reduced the induced- gelatinolytic MMP activity that was independent of TIMP-1 regulation.

### mTOR inhibitors inhibit invasion by regulating NFκB and PKC-α

**Figure 6:**
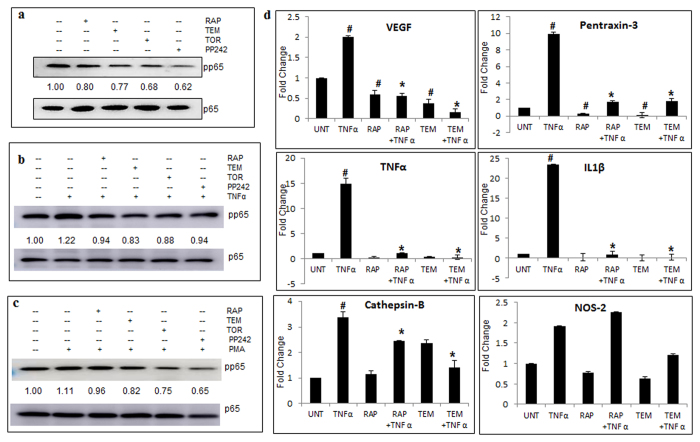
mTOR inhibitors regulate invasion by involvement of NFκB. Protein levels of phospho p65 and total p65 were measured by immunoblotting of total cell lysates of LN-18 cells treated with rapamycin-RAP (10 μM), temisirolimus-TEM (5 μM), torin-TOR (100 nM), PP-242 (100 nM) for 24 h alone and in combination with TNFα (10 ng/ml) for 12 h or PMA (100 ng/ml) for 24 h before termination of time point. Representative cropped images of three independent experiments of treatments (**a**) inhibitors alone (**b**) TNFα in combination with inhibitors (**c**) PMA in combination with inhibitors. Images show fold change of treated phospho p65 protein expression (normalised with respect to total p65) relative to untreated samples obtained by densitometry through ImageJ analysis. Full length blots are included in supplementary Fig. S12. LN-18 cells treated using RAP (10 μM) or TEM (5 μM) for 24 h alone and in combination with TNFα (10 ng/ml) for 12 h before termination of time point and real-time PCR were performed for NFκB targets VEGF, pentraxin-3, TNFα, IL1β, cathepsin-B and NOS-2. GAPDH was used as constitutionally active internal control. (**d**) The graphs represent fold change in m-RNA level of treated cells relative to untreated cells. m-RNA expression mean +/− SEM of two similar experiments performed in triplicates. ^#^p-value < 0.05 Untreated vs. TNFα or inhibitor treated; *p-value < 0.05 TNFα treatment vs. TNFα in combination with inhibitors.

We next sought to examine the mechanism involved in the action of mTOR inhibitors. NFκB is a major transcription factor that induces /elevates invasion in various cancer cells including GBM^31^. NFκB is regulated by Akt/PKB which is downstream to mTORC2^32^. In this study, immunoblotting experiments revealed that the level of constitutively expressed phospho p65 (Fig. 6a), TNFα- induced phospho p65 (Fig. 6b) were inhibited by the mTOR inhibitors. The mTOR inhibitors except for RAP reduced PMA- induced phospho p65 (Fig. 6c). Furthermore, immunofluorescence analysis supported the data demonstrating a decrease in nuclear phospho p65 by inhibitors in the presence of TNFα and PMA (Supplementary Fig. S7).

To further substantiate this data, NFκB targets which act as distinctive invasive factors were assessed. Vascular Endothelial Growth Factor (VEGF) promotes angiogenesis, pentraxin-3 is a pattern recognition molecule mediating inflammatory responses, cathepsin-B protease is often linked to tumor invasion and metastasis while Nitric oxide synthase 2 (inducible NOS-2) causes inflammation by synthesis of reactive free radical Nitric Oxide (NO). As expected, TNFα enhanced the mRNA levels of NFκB targets: VEGF (2 fold), pentraxin-3 (10 fold), TNFα (15 fold), IL-1β (23.5 fold), cathepsin-B (3.5 fold) and NOS-2 (2 fold) and the mTOR inhibitors significantly decreased the expression to ≤1 fold (p < 0.05) in all targets except cathepsin-B and NOS-2 which were marginally reduced (Fig. 6d).

**Figure 7:**
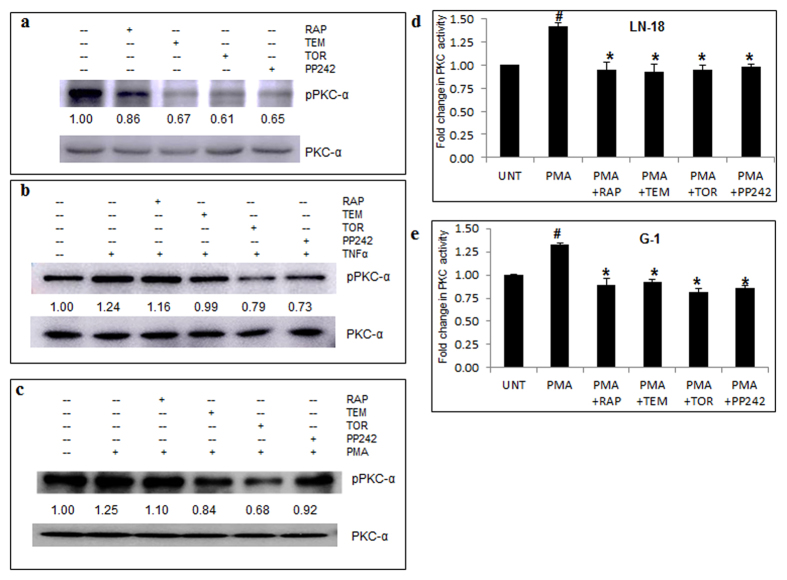
mTOR inhibitors lower PKC-α activity. Protein levels of phospho PKC-α and total PKC-α were measured by immunoblotting of total cell lysates of LN-18 cells treated with rapamycin-RAP (10 μM), temisirolimus-TEM (5 μM), torin-TOR (100 nM), PP-242 (100 nM) for 24 h alone and in combination with TNFα (10 ng/ml) for 12 h or PMA (100 ng/ml) for 24 h before termination of time point. Representative cropped images of three independent experiments of treatments (**a**) inhibitors alone (**b**) TNFα in combination with inhibitors (**c**) PMA in combination with inhibitors. Images show fold change of treated phospho PKC-α protein expression (normalised with respect to total PKC-α) relative to untreated samples obtained by densitometry through ImageJ analysis. Full length blots are included in supplementary Fig. S13. PKC Kinase activity assay was performed on whole cell lysates from cells treated with RAP (10 μM), TEM (5 μM), TOR (100 nM), PP-242 (100 nM) for 24 h in combination with PMA (100 ng/ml) of (**d**) LN-18 and (**e**) G-1 cells. The graphs represent fold change in PKC activity +/− SEM of two similar experiments performed in triplicates. ^#^p-value < 0.05 Untreated vs. PMA treated; *p-value < 0.05 PMA treatment vs. PMA in combination with inhibitors.

Another factor that is strongly associated with GBM is hyperactivation of protein kinase C alpha (PKC-α), a serine/threonine kinase and a member of the conventional (classical) PKCs. PKC-α is a target of mTORC2^33^ and plays a role in actin cytoskeleton alterations and positively regulates MMP-2. Western blotting experiments showed that mTOR inhibitors except RAP downregulated the basal levels of phospho PKC-α (Fig. 7a), as well as PKC-α activated by TNFα (Fig. 7b) and PMA (Fig. 7c). These results were confirmed by measuring pan-PKC activity in total cell lysates. PMA treatment increased the protein activity to 1.4 fold in LN-18 cells (Fig. 7d) and 1.3 fold in G-1 cells (Fig. 7e) which was restored to basal level by all the mTOR inhibitors (p ≤ 0.05).

To ascertain the role of NFκB and PKC-α during GBM invasive potential, experiments were performed to assess the effect of NFκB and PKC inhibitors on MMPs activity. 5-Aminosalicylic acid (ASA) and BAY-11 (BAY) are anti-inflammatory agents which irreversibly inhibit inducible IκBα and disrupt cytokine stimulated NFκB activation.UCN-01 (UCN) and staurosporin (STS) are ATP site binding non-selective pan PKC inhibitors. TNFα and PMA- induced MMP-9, constitutively expressed MMP-2 and PMA induced- MMP-2 were remarkably reduced by NFκB and PKC inhibitors in LN-18 (Supplementary Fig. S8a,c). In G-1 cells, TNFα induced- MMP-9 was decreased by NFκB inhibitors (Supplementary Fig. S8b) and PMA induced- MMP-2 and -MMP-9 was decreased by PKC inhibitors (Supplementary Fig. S8d). The outcome from these experiments clearly suggests the involvement of NFκB and PKC-α in the action of mTOR inhibitors to reduce the invasive potential of GBM cells. The effect of these inhibitors on cell survival was measured by MTT test. UCN showed 30% cytotoxicity at 500 nM and STS showed 50% cytotoxicity at concentration as low as 6.25 nM. ASA and BAY showed 30% cytotoxicity at 25 μM and 5 μM respectively (Supplementary Fig. S9).

### mTOR inhibitors decrease cell motility by regulating paxillin and F-actin levels

**Figure 8:**
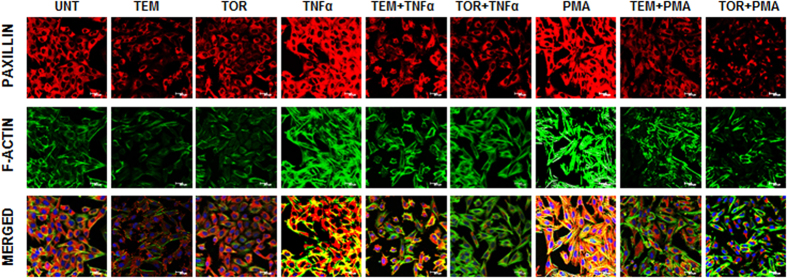
mTOR inhibitors decrease cell motility through regulating paxillin and F-actin levels. LN-18 cells seeded on coverslips were exposed to temisirolimus-TEM (5 μM) and torin-TOR (100 nM) for 24 h alone and in combination with TNFα (10 ng/ml) for 12 h or PMA (100 ng/ml) for 24 h before termination of time point were used for immunofluorescence staining of paxillin (Cy3, red) and F-actin (phalloidin, green). The merged images depict nuclear staining as blue (DAPI). Representative images of two independent experiments. Scale: 20 μm.

Paxillin is a member of focal adhesion proteins which occurs at low levels in brain tissue. Cross-talk between paxillin and actin fibers causes reorganization of cytoskeletal networks^34^. To examine whether paxillin associated with actin is regulated by mTOR inhibitors, dual immunofluorescence staining was performed. As depicted in Fig. 8, mTOR inhibitors reduced constitutive, as well as TNFα and PMA -induced paxillin levels effectively. Actin cytoskeletal regulation was also observed in accordance with paxillin levels.

## Discussion

PI3K/Akt and Ras-ERK pathways are aberrantly activated pathways in GBM. Though activated by different stimuli, both these pathways mutually regulate one another and modulate downstream targets which also include mTOR signaling. Hyperactivation of mTOR signaling is reported in glioblastoma thus making it an interesting target for therapeutic intervention^35^. Preclinical and clinical studies with mTOR inhibitors such as RAP and TEM have provided encouraging results that are largely limited to survival, cytotoxicity and antiproliferative activities^36^. However, the impact of these inhibitors in the presence of factors that contribute to invasiveness of tumors remain to be unravelled.

The microenvironment of solid tumors comprises of hyper-reactive stroma abundant in inflammatory mediators and leukocytes, dysregulated vessels and proteolytic enzymes. Also, the tumor associated macrophages (TAM) contribute to tumor progression by interaction with tumor cells and through secretion of various factors that affect angiogenesis, matrix turn over, which ultimately promote tumor invasion^37^. Together, various multiple factors activate different signaling pathways that drive the tumor cells towards more invasive and aggressive phenotypes that lead to drug resistance and recurrence resulting in poor prognosis in GBM.

TNFα, an inflammatory cytokine causes hyperactivation of the NFκB signaling pathway which results in activation of pro-survival pathway and promotes aggressive phenotype in tumor cells^38^. NFκB is a master regulator of cancer-related inflammation in TAMs and neoplastic cells. Constitutive activation of NFκB in tumors may be the result of stimulation by cytokines such as IL1β, IL-6 secreted by TAMs and other activated cells in the tumor environment^39^ or by environmental cues (e.g. hypoxia and ROI) or by genetic aberrations. NFκB induces several cellular modifications associated with tumorigenesis and more aggressive phenotypes, including self-sufficiency in growth signals, insensitivity to growth inhibition, and resistance to apoptotic signals, angiogenesis, migration and tissue invasion^40^. Phorbol-12-myristate-13-acetate (PMA) is a specific agonist of the protein kinase C (PKC) isoenzymes and a potent tumor promoter^41^. Several studies have reported high PKC activity in high grade gliomas and also provided evidence for a close relationship between PKC-α expression and invasion and migration of malignant glioma cells^42^. Recent studies demonstrated that PMA stimulated formation of invadopodia in cancer associated fibroblasts that was mediated by PKC^43^. TNFα and PMA have been used in *in vitro* experiments to study signaling pathways crucial in inducing aggressive phenotype with enhanced invasiveness. In this premise, the present study aimed to evaluate the effect of mTOR inhibitors in human GBM cells exposed to TNFα and PMA. Our results showed that despite the specificity of action, the inhibitors significantly reduced invasion and migration enhanced by TNFα and PMA in human GBM cell line and primary cultures derived from GBM tumor.

The matrix metalloproteinases, MMP-9 and MMP-2 function as key mediators of basement membrane degradation, angiogenesis, tumor invasion in GBM. Elevated level of MMP-9 is documented in glioblastoma and silencing of MMP-9 inhibits tumor invasion^44^. TNFα modulates MMP-9 expression through the classical NFκB activation and also through Ras/ERK signaling pathway by activating the NFκB and AP-1cis-elements of gene promoter binding regulatory sites^27^. MMP-9 expression is also strongly stimulated by PMA via PKC-α upstream to Ras/ERK signaling in several systems^45^. We earlier reported that silencing of rictor reduced Akt (Ser473) phosphorylation, which in turn activated Raf 1-MEK-ERK pathway leading to enhanced MMP-9 expression and activity in GBM cells^46^. Other studies demonstrated that IGF-I causes upregulation of MMP-2 synthesis via PI 3-kinase/Akt/mTOR signaling while simultaneously regulating the Raf/ERK pathway negatively^47^. PKC-α regulates many targets including MMP-9 of Ras/ ERK1/2 pathway involved in invasion of various cancers^48^ including GBM^49^. In the current study, we found that mTOR inhibitors effectively reduced the transcript and protein level of MMP-9 and MMP-2. More importantly, the functional activity of these gelatinases that was robustly induced/ enhanced by TNFα and PMA was inhibited by the mTOR inhibitors. The delicate balance between the activities of MMPs and Tissue inhibitor of metalloproteases (TIMPs) is critical to limit deleterious outcomes of uncontrolled degradation which is manifested in tumor cell invasion and angiogenesis mediated by inflammatory cytokines^25^,^50^. Some reports suggest a correlation between reduced expression of TIMP-1 and -2 with increasing glioma grade predicting that a lack of inhibitor expression may contribute to a more aggressive glioma phenotype^51^ while other studies reported upregulation of TIMP-1 or -2 expressions in invasive malignant tumors^52^. Our results revealed that MMP-9 activity reduced by mTOR inhibitors was independent of TIMP1. Collectively, the findings suggest that the inhibitors targeting predominantly mTORC1 (RAP and TEM) as well as inhibitors to both C1 and C2 (TOR and PP242) were effective in reducing the invasion, migration and MMP activity induced by TNFα and PMA.

To decipher the mechanism involved in inhibiting the invasiveness and modulating the MMP activity, we examined the impact of the mTOR inhibitors on NFκB and PKC-α -mediated signaling. The mTOR inhibitors - TEM and TOR restored the level of phospho p65 and PKC-α activity induced by TNFα and PMA. The transcripts of VEGF, pentraxin-3, genes associated with invasion and migration were greatly reduced by mTOR inhibitors. In this context, it is noteworthy that interaction between VEGF and growth factor receptors lead to evasive anti-angiogenic drug resistance and currently studies involving combination therapy using anti-VEGF and PI3K/Akt/mTOR inhibitors are being pursued as therapeutic options^53^,^54^. A recent report underscores the role of mTORC2, that is independent of Akt/mTORC1 during regulation of angiogenesis through regulation of Extra Cellular focal adhesion kinase activity, matrix adhesion, and cytoskeletal remodelling^55^. Pentraxin-3 is positively correlated with tumor grade and severity and is emerging as a novel bio-marker for cancer-related inflammation in various cancers including glioma^56^.

Cell migration and motility is regulated by integration and dissemination of signals from integrins and growth factor receptors. Paxillin is regarded as critical downstream target of integrins and modulates the proper formation of focal adhesion complex involved in PMA-stimulated migration^57^. Paxillin is present in low levels in normal brain tissue and elevated in many cancers with the levels correlating with higher invasive potential and migration. Paxillin is regarded as a potential biomarker as it negatively correlates with patient survival. A recent report suggested that IGF-I-induced F-actin reorganization and phosphorylation of focal adhesion proteins were inhibited by disruption of mTOR-raptor complex by RAP^58^. A recent study showed that in GBM, mTORC2 plays important role in cell motility and invasion by association with Filamin A (FLNA) which is a widely expressed protein that regulates reorganization of the actin cytoskeleton^59^. On these lines, it is interesting to note that TEM and TOR reduced the expression of paxillin strengthening the impact of these inhibitors as effective anti-invasive treatments.

Since our findings demonstrated that mTOR inhibitors targeted both NFκB and PKC-α, we confirmed the role of NFκB and PKC-α downstream signaling during tumor invasion. We showed that NFκB inhibitors-5-Aminosalicylic acid (ASA) and BAY-11 (BAY) and PKC inhibitors-UCN-01 (UCN) and staurosporin (STS) effectively reduced MMP activity in LN-18 and G-1 cells confirming the role of NFκB and PKC-α signaling during tumor invasion. UCN-01 is a pan-PMA inhibitor used in combination therapies during preliminary phases of clinical trials for various cancers^60^. These inhibitors reduced MMP activity; however the concentrations at which they were effective were toxic to the cells suggesting that the effect could be due to cell death. In contrast, mTOR inhibitors -TEM and TOR were non-toxic at concentrations that were effective in reducing invasiveness suggesting their targeted action in glioma cells. Though these results point to the involvement of NFκB and PKC-α, we cannot rule out the possibility of the mTOR inhibitors modulating invasion via other signaling pathways stimulated by TNFα and PMA.

New concepts are underway for designing novel therapeutic approaches to improve prognosis in recurrent glioblastoma. These approaches involve development of a regime with a combination of drugs not traditionally thought of as cytotoxic chemotherapy agents but that have a robust history of being well-tolerated and are already marketed and used for other non-cancer indications^61^. Based on these evolving concepts, our study suggests that mTOR inhibitors such as TEM have a high therapeutic value in treatment of malignant gliomas for the following reasons: i) TEM and TOR control tumor progression by reducing invasion, migration and MMP activity, ii) inhibitors are effective by inhibiting NFκB and PKC-α signaling pathways that are crucial for tumor progression, iii) TEM is already in use for treatment of various cancers in combination with other drugs and has the advantage of being able to cross the blood brain barrier, iv) as TMZ (first drug of choice) improves GBM patient survival only by ~11% and patients develop resistance to TMZ, TEM is a reasonable option for reducing aggressiveness and improving susceptibility to chemotherapy. Lastly, considering that low grade gliomas have a propensity to be driven to a more aggressive phenotype through signaling from the microenvironment, TEM and other mTOR inhibitors can be explored in combination with other drugs for better and effective treatment regime in such tumors.

## Materials and Methods

### Cell Lines and Primary Cell Cultures

The Human Glioblastoma Cell line LN-18 was obtained from American Type Culture Collection (ATCC Rockville, USA). The Human Glioblastoma tumor tissue samples were collected from surgeries performed at Sasoon hospital, DY Patil Hospital and Inamdar hospital, Pune. Informed consent was obtained from patients for tissue procurement in accordance with the protocol approved by the institutional ethics committee of NCCS and graded by pathologist. Primary Cultures were obtained by processing GBM tumor samples using Accutase (Himedia) and Zymefree (Himedia) to obtain adherent cell cultures which were passaged to 3–10 passages. We have previously reported the expression of neuronal markers in primary cultures-G1^62^. Cells were maintained in Dulbecco’s modified eagle’s medium (DMEM) with 4 mM L-glutamine, 1.5 g/L sodium bicarbonate and 4.5 g/L glucose, supplemented with 5% heat inactivated fetal calf serum (Gibco) in a humidified incubator at 37 °C with 5% CO_2_. Cells were dislodged using trypsin (0.125%) – EDTA (0.02%) solution.

### Treatment

Cells were treated with mTOR inhibitors- Rapamycin (RAP) (10 μM) (Calbiochem) or Temisirolimus (TEM) (5 μM) (Santacruz biotechnology) or Torin-1 (TOR) (100 nM) (Tocris) or PP242 (100 nM) (Tocris) and cytokines- Tumor Necrosis Factor-α (TNFα) (10 ng/ml) (Peprotech), Interleukin1β (IL1β) (10 ng/ml) (Peprotech) and tumor promoting compound Phorbol 12- myristate 13-acetate (PMA) (100 ng/ml) (Sigma Aldrich). Staurosporin (STS) (50 nM) and UCN01 (500 nM) (Sigma Aldrich) are pan-inhibitors of PKC kinases. Amino salicylic acid (ASA) (20 μM) and BAY11 (10 μM) (Sigma Aldrich) are specific NFκB inhibitors. Experiments were performed on cells treated in complete medium except for gelatinolytic zymography, invasion and migration assays where serum-free medium was used.

### Cell Viability Assay

Dose and time dependent cell viability assay was carried out with LN-18 cells and G1 cells using MTT (3-(4, 5-dimethylthiazole-2-yl)-2, 5-diphenyltetrazolium bromide) (Sigma Aldrich). 5 × 10^3^ cells were grown in 96 well plates for 24 h to obtain 80% confluency and then treated using fresh medium with serial concentrations of RAP, TEM, TOR and PP242 for 24 h, 48 h or 72 h time periods. The effect of mTOR inhibitors in “washout” experiments was assessed in G-1 cells treated with RAP (10 μM), TEM (5 μM), TOR (100 nM) or PP-242 (100 nM) for 24 or 48 h. The media containing inhibitor was aspirated and cells were washed with media and replenished with inhibitor-free fresh complete media and the cells were further incubated for 24 h or 48 h respectively. Another set of treated cells was maintained without “washout” for 72 h time period and was regarded as control set. To terminate the experiments, control and test samples were incubated with 0.5 mg/ml of MTT in PBS for 4 h. The formazan crystals formed were dissolved using 10% SDS and absorbance was measured at 570–640 nm using microplate reader (Spectromax 250, Molecular Devices). The percentage of viable cell count was calculated assuming control viable cell count as 100%.

### RNA Isolation, Semi-quantitative PCR and Real-Time PCR Analysis

RNA isolation from cells was performed using Trizol reagent (Invitrogen) and c-DNA was synthesized by Improm II reverse transcriptase system (Promega). The absorbance at 260 and 280 nm was measured using NanoDrop ND-1000 UV-Visible Spectrophotometer. A260/A280 ratio of 1.9 to 2.1 indicated good quality of RNA and c-DNA.

Quantitative real time PCR was performed using SYBR Green Supermix (Biorad) in Realplex Real-Time Thermal Cycler (Eppendorf). The profile of thermal cycling consisted of initial denaturation at 95 °C for 2 min, and 40 cycles at 95 °C for 15 s and 60 °C for 45 s for primer annealing and extension. Melting curve analysis was used to determine the specific PCR products. The changes in the threshold cycle (C_T_) values were calculated by the equation: - ∆C_T_ = C_T (target gene)_ - C_T (endogenous control gene)_ and fold difference was calculated as 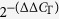. C_T_ values and melting curves were analyzed on Eppendorf Realplex 2.2 software. GAPDH was used as an internal control to normalize gene expression. Fold change for each treated sample was calculated in comparison with constitutive m-RNA levels of the specific gene and graphs were plotted. Sequences of primers used in the study are listed in the Supplemental Table 1.

### Western blotting

Cells were harvested and lysed using RIPA lysis buffer [120 mM NaCl, 1.0% Triton X-100, 20 mM Tris–HCl, pH 7.5, 100% glycerol, 2 mM EDTA and protease inhibitor cocktail, (Roche). Bradford method (Biorad) was used to estimate total protein concentration. Total protein (35 μg) of each sample was electrophoresed on 10% SDS polyacrylamide gels at constant voltage of 65 V and electro-blotted onto PVDF membrane (Millipore) using Bio-Rad mini-blot module (120 mA per gel, 3 h, and 4 °C). After blocking with 5% BSA in TBS-T buffer for 1 h at room temperature, the blots were probed with specific primary antibodies for 2 h at room temperature or overnight at 4 °C. Phospho p65 (Ser276) (1:2000), total p65 (1:2000), Phospho Akt (Ser473) (1:2000), total Akt (1:2000) and total PKC-α (1:2000) primary antibodies were from Santacruz Biotechnology. Phospho PKC-α (Ser657 +Tyr658) (1:1000) was from Abcam and Phospho mTOR (Ser2448) (1:1000), total mTOR (1:1000), S6kinase (Thr389) (1:1000) and total S6kinase (1:1000) were from Cell signaling technology. HRP-labelled secondary antibodies anti-rabbit or anti-goat (1:8000) (Biorad) were probed for 1 h at room temperature. The bands were visualized by chemiluminescence using Super Signal West Femto Maximum Sensitivity Substrate (Pierce) and images were acquired on Amersham Imager 600 instrument (General Electric, GE). GAPDH (Sigma Aldrich, 1:10,000) was used as loading controls. For analysis of relative intensities of protein, densitometry was performed using the ImageJ software.

### Enzyme Linked Immunosorbent Assay (ELISA)

Amersham Human MMP-2 Biotrak ELISA system (GE Health Care) was used according to manufactures protocol to quantify protein concentrations of MMP-2 in conditioned media. Absorbance was measured at 450 nm using micro-plate reader (Spectromax 250, Molecular Devices). Unknown protein concentrations of samples were calculated from linear regression equation of standard graph.

### PKC kinase activity assay

PKC kinase activity was determined using colorimetric assay kit according to the manufacturer’s instructions (Enzo life sciences). Sample lysates, standards were added onto substrate coated assay plate along with ATP and incubated for 90 min at 30 °C (Reaction initiation). Wells were emptied (Reaction termination), Phospho specific substrate antibody was added and incubated for 60 min at room temperature. After incubation for 30 min with anti-IgG:HRP conjugate and appropriate washes TMB substrate was added and incubated for 30 min at room temperature. Stop solution was added and absorbance was measured immediately at 450 nm.

### Gelatin Zymography

Non-denatured conditional media (serum-free) were mixed with 4× sample buffer without reducing agents and resolved on 7.5% SDS-PAGE gels impregnated with 0.1% gelatin (USB). Electrophoresis was performed at 65 volts (constant voltage) and gels were washed twice (30 min/wash) with washing buffer (50 mM Tris-Cl, pH 7.5 and 2.5% Triton X-100). Degrading enzymatic activity after overnight incubation in renaturation buffer (50 mM Tris-Cl, pH 7.6, 10 mM CaCl_2_, 150 mM NaCl, and 0.05% NaN_3_) was checked by band intensity on staining with 0.2% Coomassie Brilliant Blue R-250 in 40% isopropanol and destained using 7% glacial acetic acid. Zymograms were acquired in Biorad gel documentation system and analysed using Syngene software.

### Immunofluorescence staining

LN-18 and G-1 cells (5 × 10^4^ cells) grown on 22 × 22 square cover slips (Thermo Esco) in complete medium for 24 h were treated using mTOR inhibitors in the presence or absence of TNFα and PMA for specific periods. Cells were washed twice using cold 1 × PBS and fixed in 3.7% paraformaldehyde for 10 min at room temperature. Cells were then permeabilized for 10 min with freshly prepared 0.2% Triton-X 100 in PBS, blocked using 1% BSA in PBS for 1 h. The cells were incubated with optimal dilutions of primary antibodies - MMP-2 (1:200) (Alexis), MMP-9 (1:200) (Alexis), phospho p65 (1:200) (Santacruz biotechnology), TIMP-1 (1:100) (Santacruz biotechnology), paxillin (1:200) (Santacruz biotechnology), actin-phalloidin (1:100) (Molecular probes) for 2 h, followed by fluorescence Cy3 conjugated secondary antibodies anti-rabbit (1:250) (Chemicon) for 1 h. Nuclear staining was done using DAPI (0.5 μg/ml) (Invitrogen) for 30 min and cover slips were mounted using mounting media. Images were acquired using LEICA confocal microscope.

### Matrigel Invasion assay

BioCoat Matrigel Transwell chambers (8-μm polycarbonate Nucleopore filters, BD Biosciences) were used as an *in vitro* model for assessment of invasive property. Cells seeded in 6 well plates were cultured for 24 h in complete media to obtain 80% confluency and pre-treated with TNFα or PMA in combination with mTOR inhibitors for 6 h prior to adding the cells into the inserts. Invasion chambers were filled with 500 μl of serum-free DMEM containing bicarbonate and incubated in humidified incubator, 37 °C, 5% CO_2_ atmosphere for 2 h for rehydration. Conditioned complete medium, in which cells were grown for 24 h, was added to the wells (500 μl/well). TNFα or PMA was added to the conditioned medium to act as chemo-attractant to the cells. Cell suspension of each treatment, containing 2.5 × 10^4^ cells was prepared in 500 μl of serum-free medium which was added to the inserts and chambers were incubated for 22 h in humidified CO_2_ incubator. The wells and inserts were washed with 1× PBS and fixed using 4% paraformaldehyde (PFA) for 15 min at room temperature. The non-invasive cells from the inside surface of the insert were scrubbed using cotton buds and washed with 1 × PBS. The invaded cells adhered to the outer surface of the insert were stained with 0.2% crystal violet in 2% ethanol for 20 min. The images were acquired using phase- contrast microscope (Nikon). Each experiment was done in duplicates and fold change was calculated from cell count per field (5 fields per treatment). Graphs and images indicate the invasive potential of the cells. Number of invasive cells were counted (5 fields) by ImageJ software, normalized and represented as the fold change (Mean ± SD).

### Migration assay

Cells (2.5 × 10^4^) were seeded in 12 well plates and 80% confluency was obtained. The monolayer of cells was pre-treated with drug actinomycin D (100 ng/ml) (Sigma Aldrich) for 3 h and then scratched with a pipette tip held at an angle of 45° to simulate wound. The medium was removed and cells were washed using fresh medium. The cells were treated with TNFα or PMA in combination with mTOR inhibitors for 16 h was performed in serum-free medium. Randomly chosen fields (n = 5) were used to capture the images at identical locations at time 0 h and 16 h using phase-contrast microscope (Nikon) under bright light. The wound closure widths were measured by ImageJ software, normalized [(width at 0 h–width at 16 h)/width at 0 h] and represented as the fold change of wound closure calculated as width of treated/width of untreated (Mean ± SD).

### Mitochondrial membrane potential

G-1 cells (2.5 × 10^4^) were seeded in 12 well plates and 80% confluency was obtained. Cells were treated with mTOR inhibitors in the presence or absence of TNFα or PMA for 16 h. Cells were dislodged using TPVG, washed in 1× PBS and incubated in JC-1 dye (2 μM) (ThermoFisher scientific) for 15 min at 37 °C in CO_2_ incubator. Stained cells were resuspended in 1× PBS and acquired using BD FACSCalibur flow cytometer. Analysis was done on Cell Quest Pro software and shift in depolarization of membrane potential (green fluorescence of JC-1 monomers) was plotted as overlay histograms.

### Statistical Analysis

Quantitative data was represented as mean ± standard error of the mean (SEM) for various experimental groups. The statistical significance between groups was analysed using an unpaired Student’s *t* test to obtain p-value. P < 0.05 was considered significant.

## Additional Information

**How to cite this article**: Chandrika, G. *et al.* Suppression of the invasive potential of Glioblastoma cells by mTOR inhibitors involves modulation of NFκB and PKC-α signaling. *Sci. Rep.* **6**, 22455; doi: 10.1038/srep22455 (2016).

## Supplementary Material

Supplementary Information
