# Supplementary material for: Suppression of the invasive potential of Glioblastoma cells by mTOR inhibitors involves modulation of NFκB and PKC-α signaling
Source: Sci Rep. 2016 Mar 4;6:22455. doi: 10.1038/srep22455 (PMC4778030; doi:10.1038/srep22455)
Supplement: Supplementary Information [file srep22455-s1.doc]

**Supplementary Figures**

**Title: Suppression of the invasive potential of Glioblastoma cells by mTOR inhibitors involves modulation of NFκB and PKC-α signaling**

**Authors:** Goparaju Chandrika^1#^, Kumar Natesh^1#^, Deepak Ranade^2^, Ashish Chugh^3^ and Padma Shastry^1^**^*^**


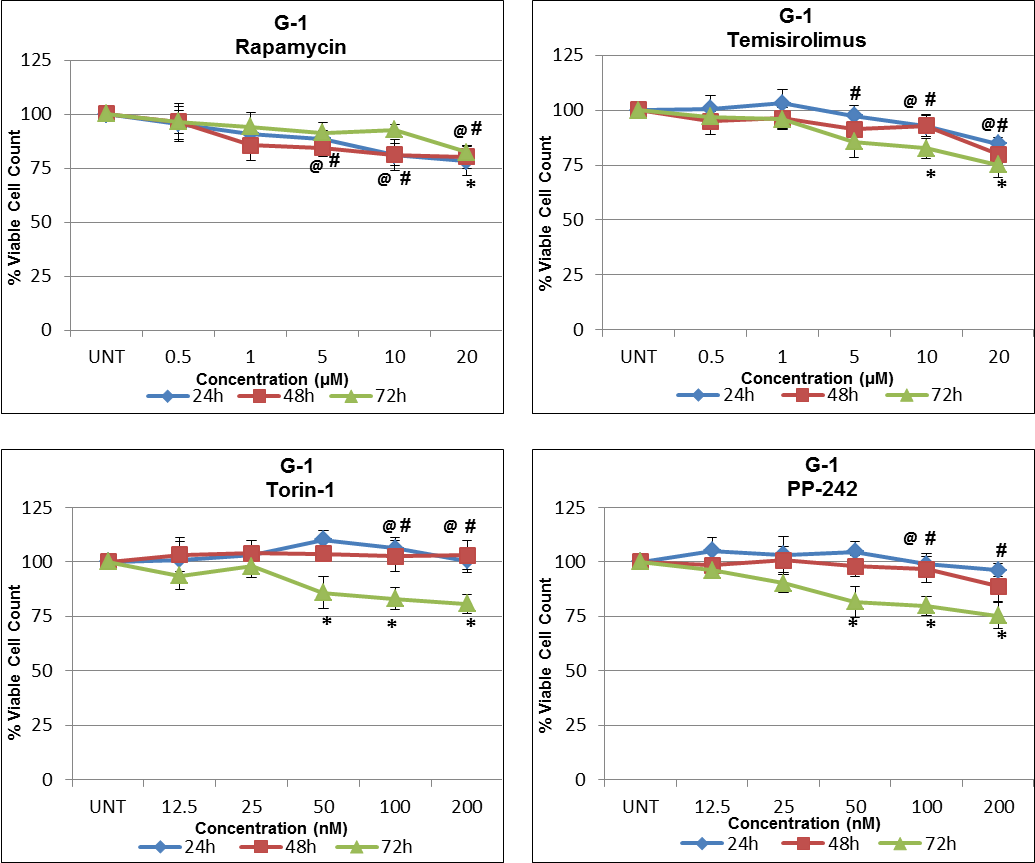


**Supplementary Figure S1.** **mTOR inhibitors decrease viable cell number in primary culture cells:** G-1 cells were treated with serial concentrations of rapamycin-RAP, temisirolimus-TEM, torin-TOR and PP-242 for 24h, 48h and 72h and percentage of viable cell count was assessed by MTT assay. Viable count of untreated cells was assumed as 100%. The graphs represent % viable cell count +/- SEM of three similar experiments performed in triplicates. @p-value <0.05 Untreated vs. inhibitor treatment for 24h; #p-value <0.05 Untreated vs. inhibitor treatment for 48h; *p –value <0.05 Untreated vs. inhibitor treatment for 72h.


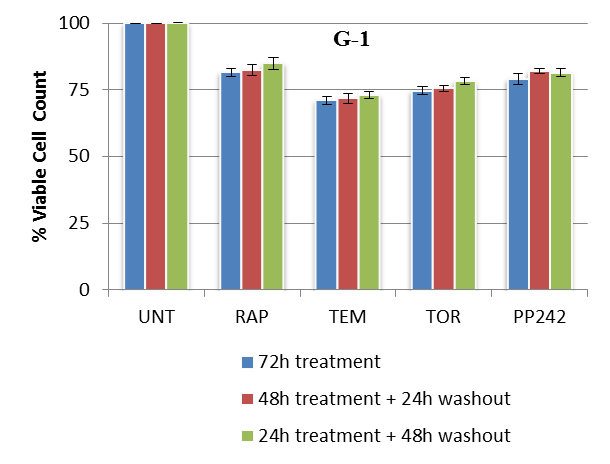


**Supplementary Figure S2. mTOR inhibitors show sustained effect on viable cell count:** MTT assay was performed to determine effect of mTOR inhibitors on cell growth during “washout” condition. G-1 cells were treated with rapamycin-RAP (10μM) or temisirolimus-TEM (5μM) or torin-TOR (100nM) or PP-242 (100nM) for 48h or 24h, then the media containing inhibitor was removed, washed with media and then fresh complete media without inhibitors was added and the cells were incubated for 24h or 48h respectively. Another set of treated cells was maintained without “washout” for 72h time period and was regarded as control set. Viable count of untreated cells was assumed as 100%. The graph represents % viable cell count +/- SEM of three similar experiments performed in triplicates.


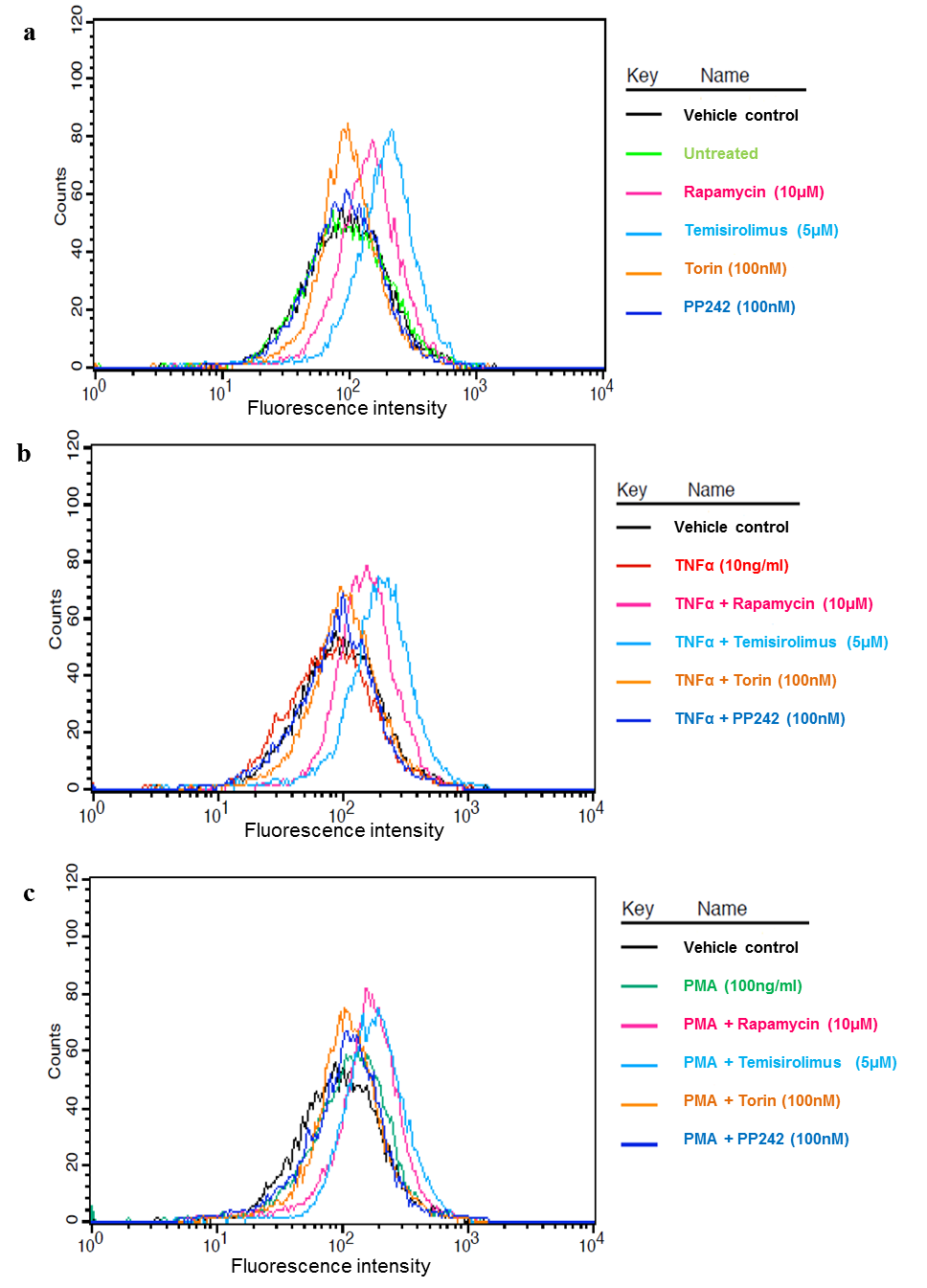


**Supplementary Figure S3.** **Effect of mTOR inhibitors on mitochondrial membrane potential:** Flow cytometric analysis of G-1 cells using JC-1 dye (2μM) was performed to assess change in mitochondrial membrane potential. Histogram overlays representing green fluorescence of JC-1 dye monomers were compared for a) Vehicle control vs. individual inhibitor treatment; b) TNFα vs. TNFα in combination with inhibitors; c) PMA vs. PMA in combination with inhibitors.


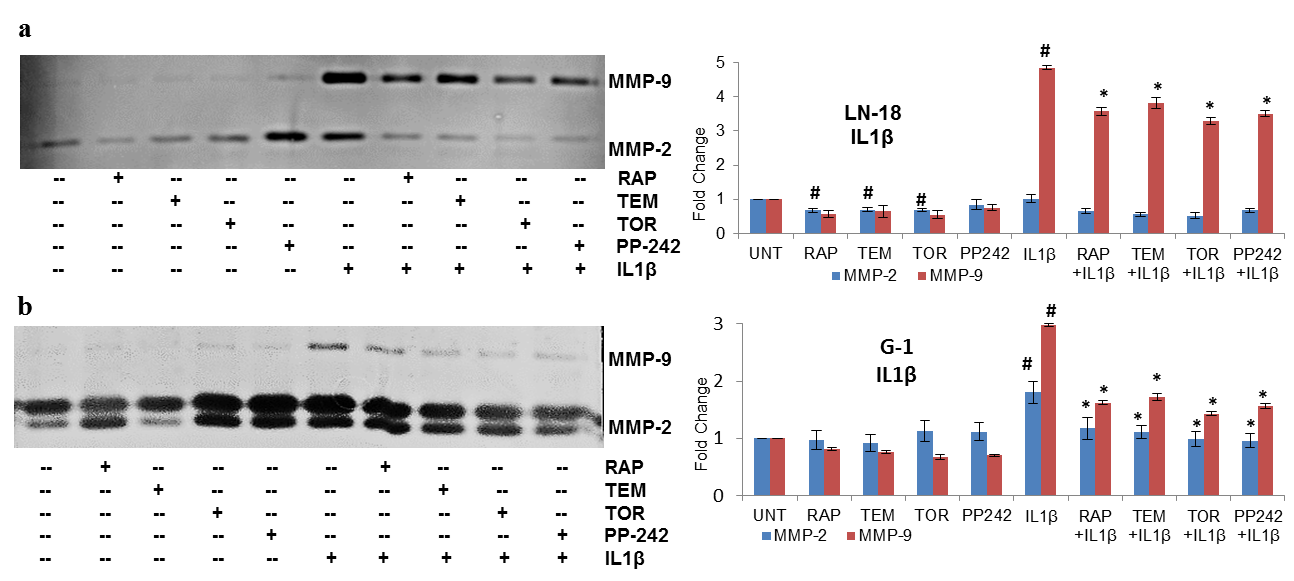


**Supplementary Figure S4.** **mTOR inhibitors revert** **IL1β induced-gelatinolytic MMP activity:** Gelatinolytic Zymography analysis for functional activity of MMP-9 ( 92KDa) and MMP-2 (72 KDa) was performed on conditional media from cells treated using rapamycin-RAP (10μM), temisirolimus-TEM (5μM), torin-TOR (100nM), PP-242 (100nM) alone and in combination with IL1β (10ng/ml) for 24h before termination of time point. Representative images of zymograms of (a) LN-18 and (b) G-1 cells. The graphs represent fold change in gelatinolytic enzyme activity +/- SEM of three independent experiments obtained by densitometry through ImageJ analysis. # p-value <0.05 Untreated vs. IL1β or inhibitor treated; *p–value <0.05 IL1β treatment vs. IL1β in combination with inhibitor treatment.

**
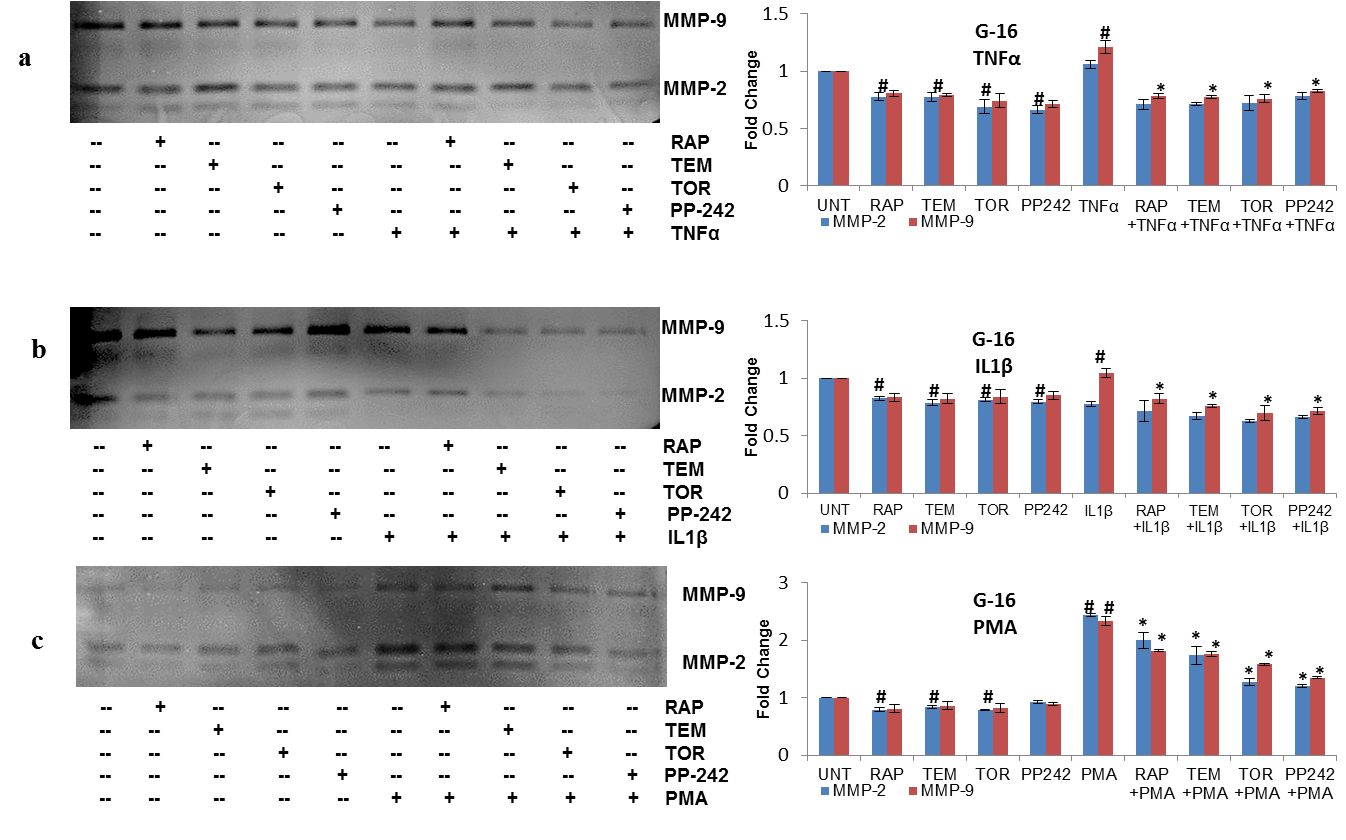
**

**Supplementary Figure S5. mTOR inhibitors revert** **induced-gelatinolytic MMP activity in GBM primary culture:** Gelatinolytic Zymography analysis for functional activity of MMP-9 (92KDa) and MMP-2 (72 KDa) was performed on conditional media from primary culture G-16 cells treated using rapamycin-RAP (10μM), temisirolimus-TEM (5μM), torin-TOR (100nM), PP-242 (100nM) alone and in combination with TNFα (10ng/ml) for 12h before termination of time point or IL1β (10ng/ml) or PMA (100ng/ml) for 24h. Representative zymogram images of G-16 treated by (a) TNFα, (b) IL1β and (c) PMA alone or in combination with inhibitors. The graphs represent fold change in gelatinolytic enzyme activity +/- SEM of two independent experiments obtained by densitometry through ImageJ analysis. # p-value <0.05 Untreated vs. TNFα or IL1β or PMA or inhibitor treated;*p–value <0.05 TNFα or IL1β or PMA treatment vs. TNFα or IL1β or PMA in combination with inhibitor treatment.

**
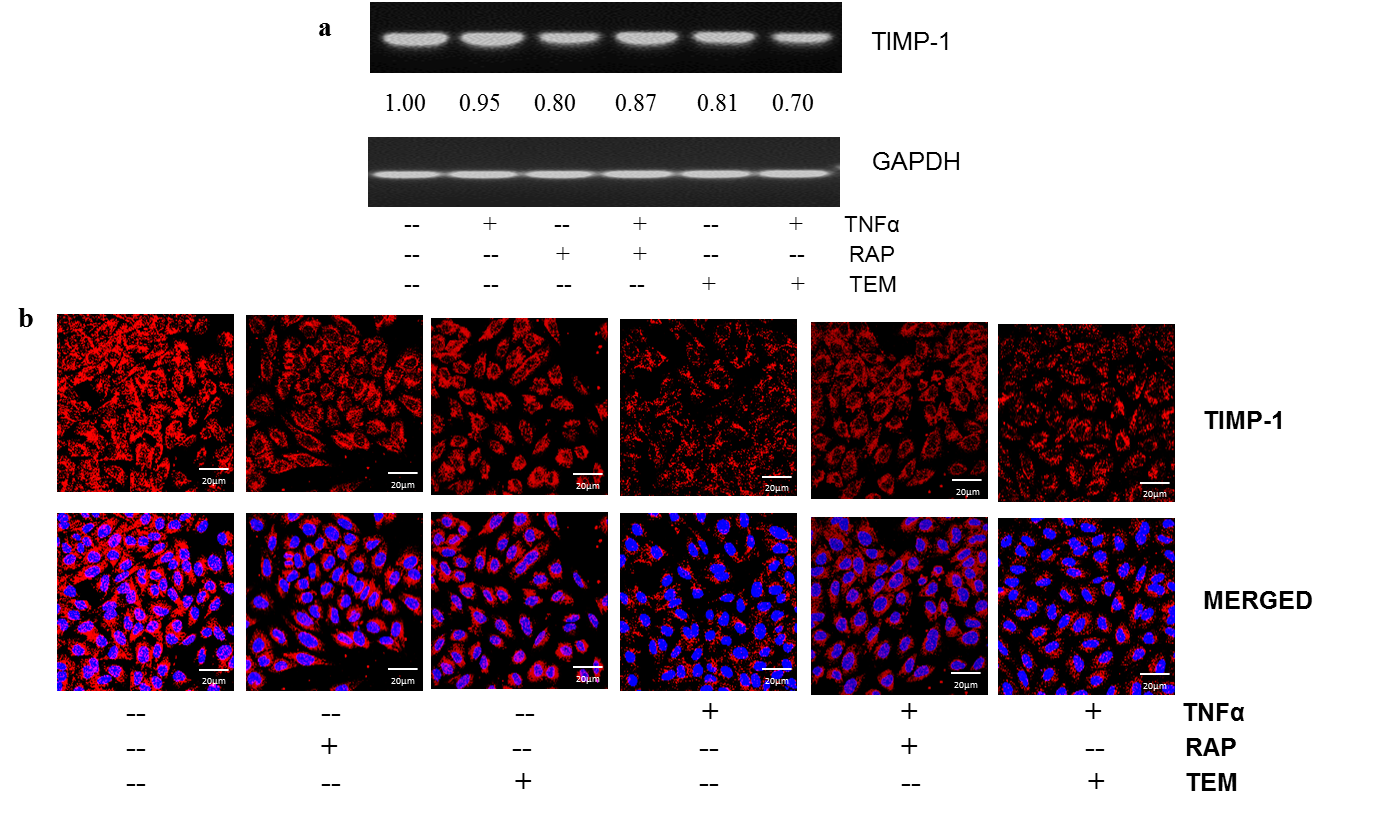
**

**Supplementary Figure S6.** **mTOR inhibitors are ineffective on TIMP-1:** LN-18 cells treated using rapamycin-RAP (10μM), temisirolimus-TEM (5μM) for 24h alone and in combination with TNFα (10ng/ml) for 12h before termination of time point were used to perform semi-quantitative PCR for m-RNA expression and immunofluorescence staining for protein intensity of TIMP-1. (a) Representative image showing fold change of TIMP-1 m-RNA expression relative to untreated cells obtained by densitometry through ImageJ analysis. GAPDH was used as constitutionally active internal control. (b) Representative images of TIMP-1 protein intensity from two independent experiments. Nuclear staining with DAPI. Scale: 20μm.


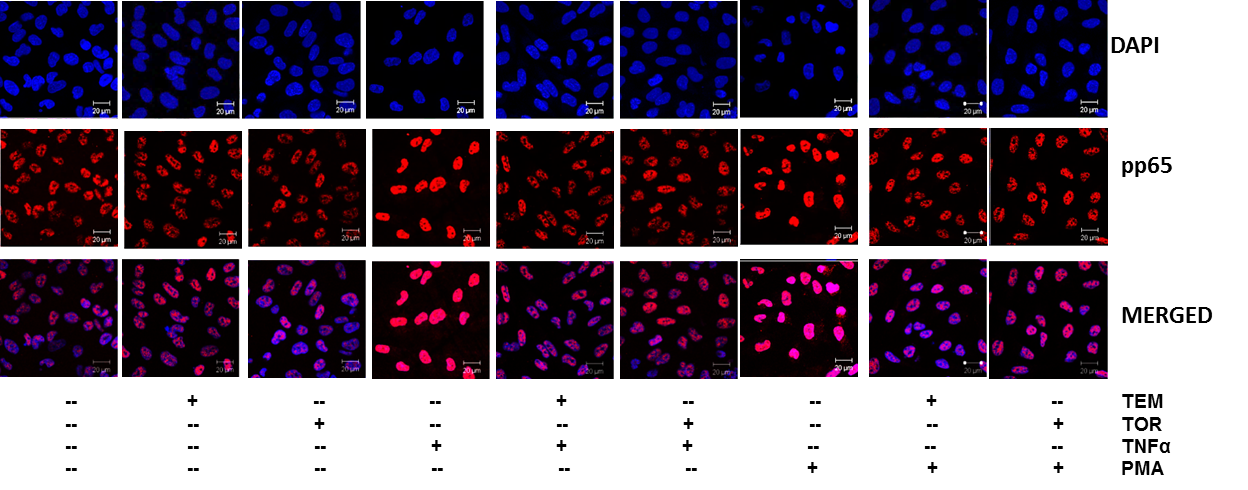


**Supplementary Figure S7. mTOR inhibitors regulate nuclear localisation of pp65:** Immunofluorescence staining for phospho p65 protein intensity was performed on LN-18 cells treated using temisirolimus-TEM (5μM) or torin-TOR (100nM) alone and in combination with TNFα (10ng/ml) for 12h or PMA (100ng/ml) for 24h before termination of time point. Nuclear staining with DAPI. Representative images of two independent experiments. Scale: 20μm.


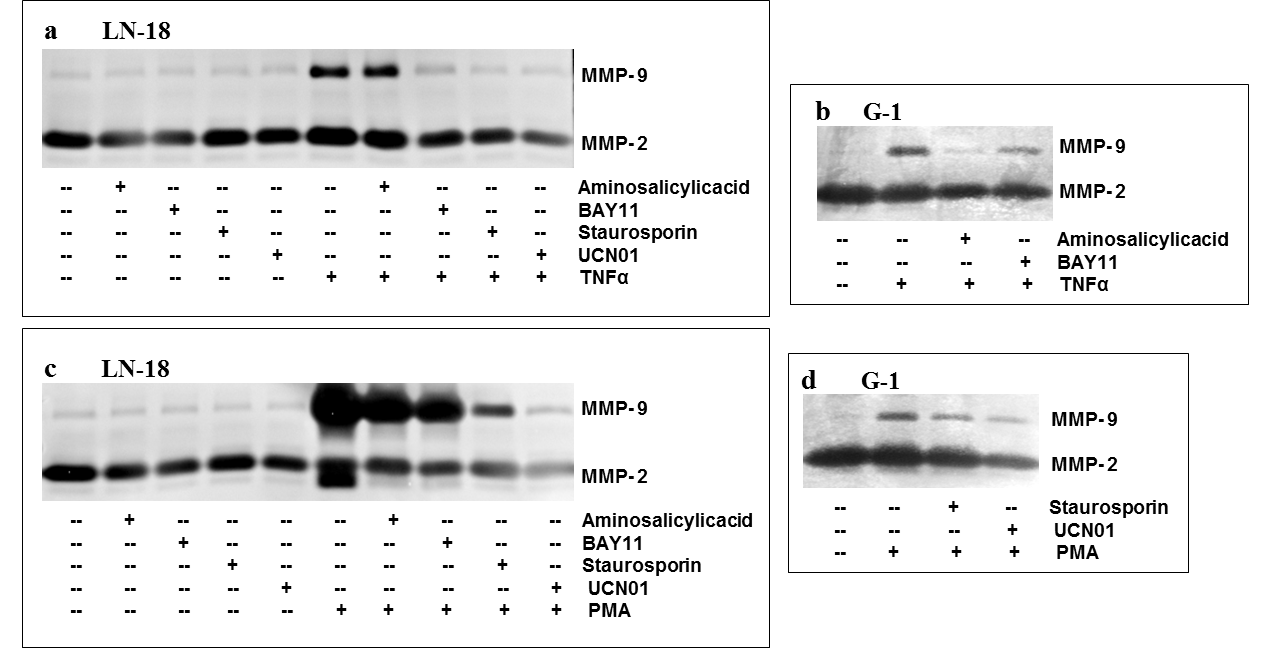


**Supplementary Figure S8.** **NFκB and PKC inhibitors reduce induced-gelatinolytic MMP activity:** Cells were treated for 24h with 5-Amino salicylicacid (20μM), BAY-11 (10μM), Staurosporin (50nM), and UCN-01 (500nM) alone and in combination with TNFα (10ng/ml) in (a) LN-18 and (b) G-1cells or with PMA (100ng/ml) in (c) LN-18 and (d) G-1 cells. Gelatin zymography was performed using conditioned media. The zymograms are representative of two independent experiments.


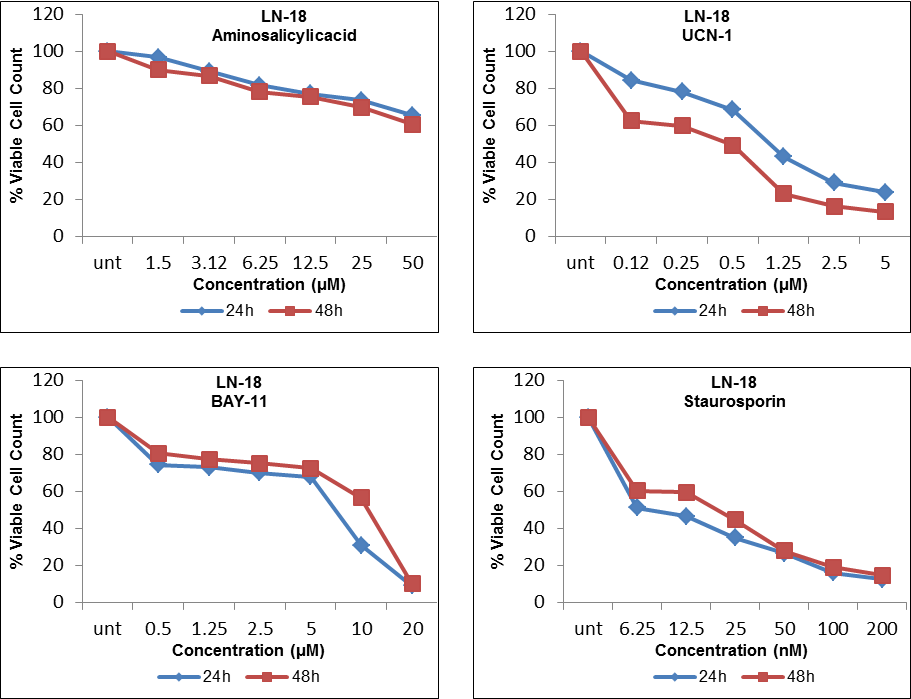


**Supplementary Figure S9. Effect of NFκB and PKC inhibitors on viable cell count:** LN-18 cells were treated with different concentrations of NFκB inhibitors**:** 5-Aminosalicylic acid (ASA) and BAY-11 (BAY) as well as PKC inhibitors: UCN-01 (UCN) and Staurosporin (STS) for 24h and 48h and cell viability by assessed by MTT assay. Cell viability of untreated cells was assumed as 100%. The graphs represent % viable cell count of two similar experiments performed in triplicates.


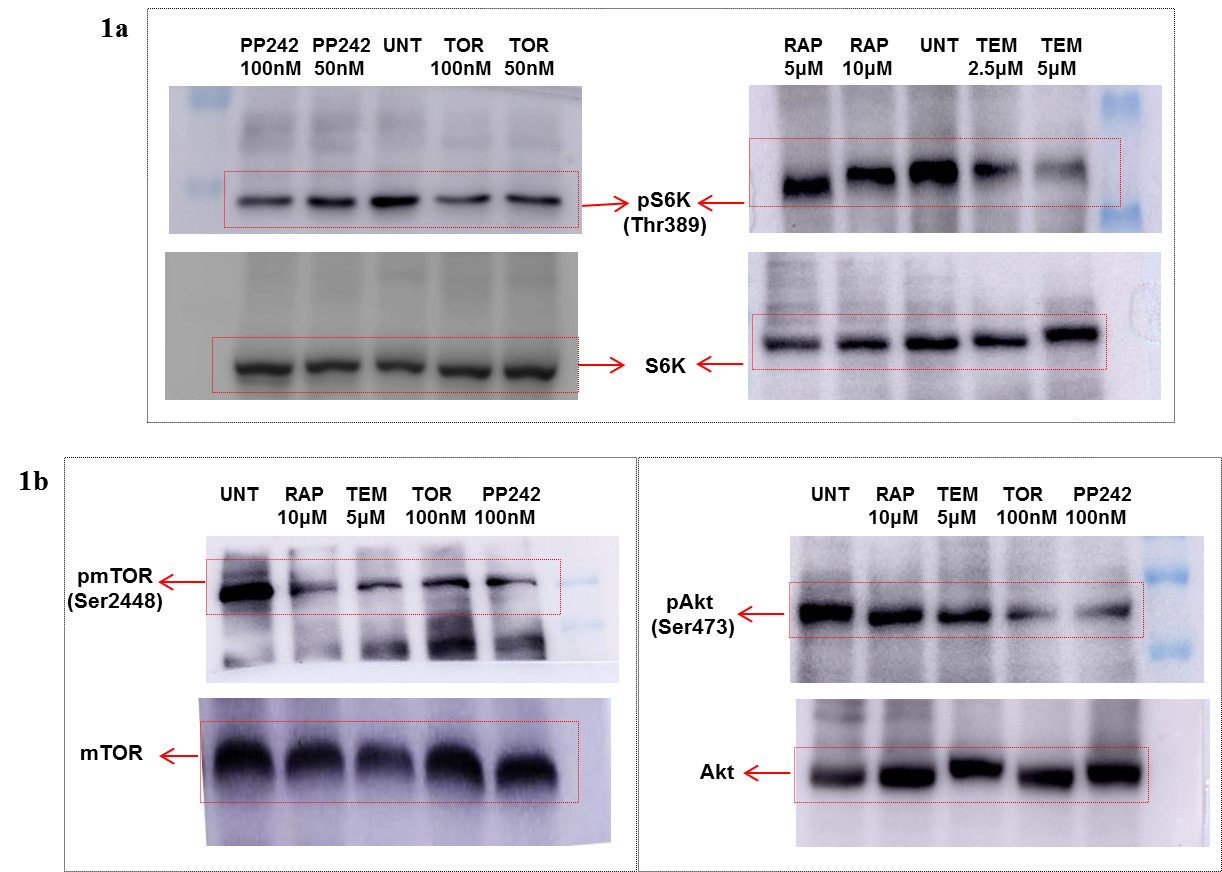


**Supplementary Figure S10.** Full-length blots relative to the cropped images showed in the main Figure 1b and 1c.


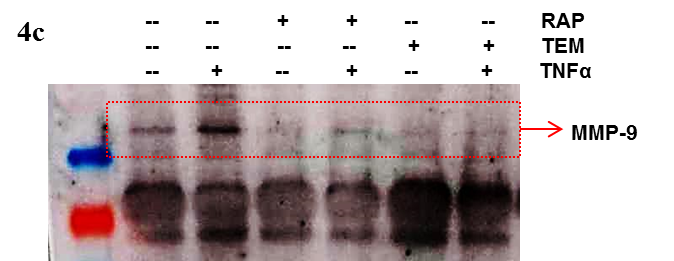


**Supplementary Figure S11.** Full-length blot relative to the cropped image showed in the main Figure 4c.


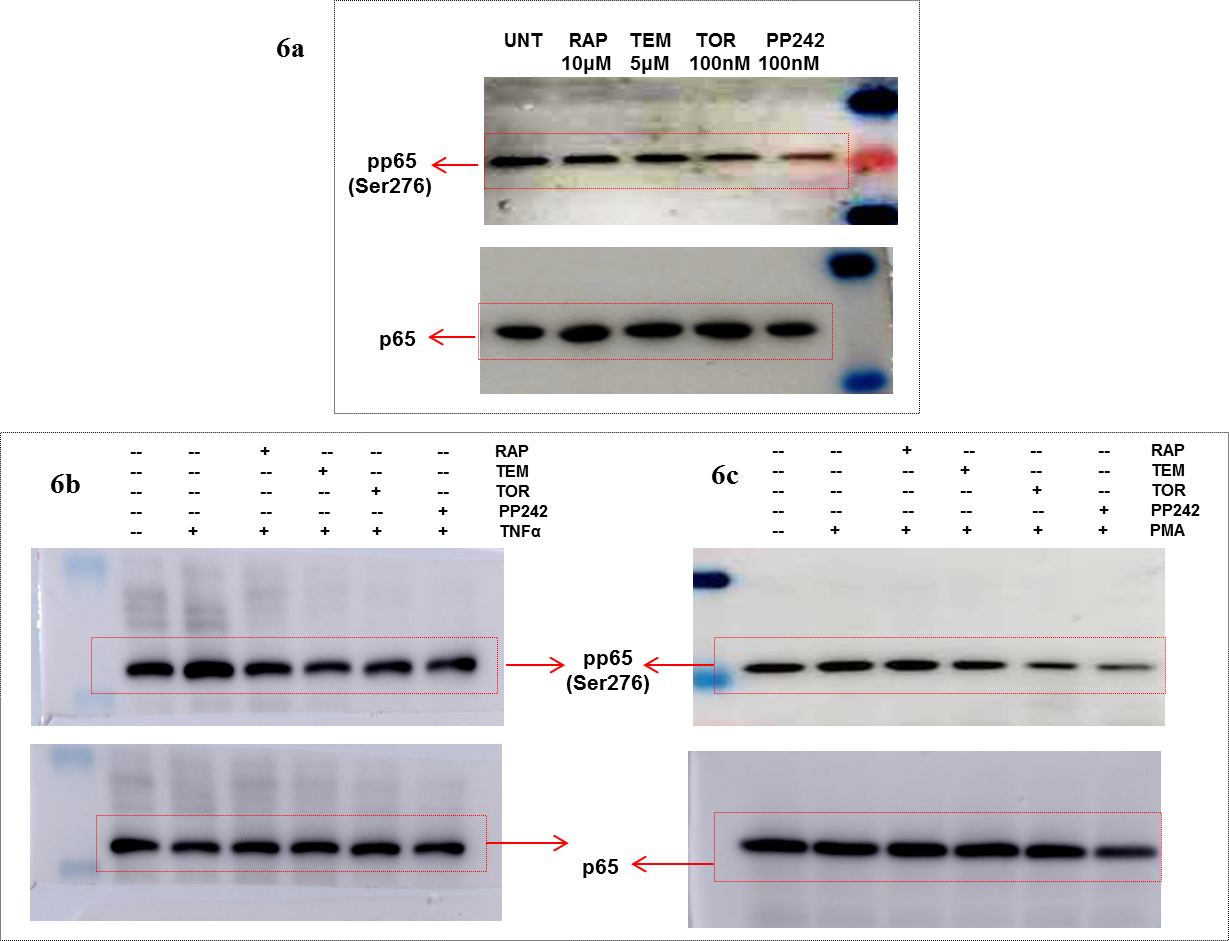


**Supplementary Figure S12.** Full-length blots relative to the cropped images showed in the main Figure 6a, 6b and 6c.


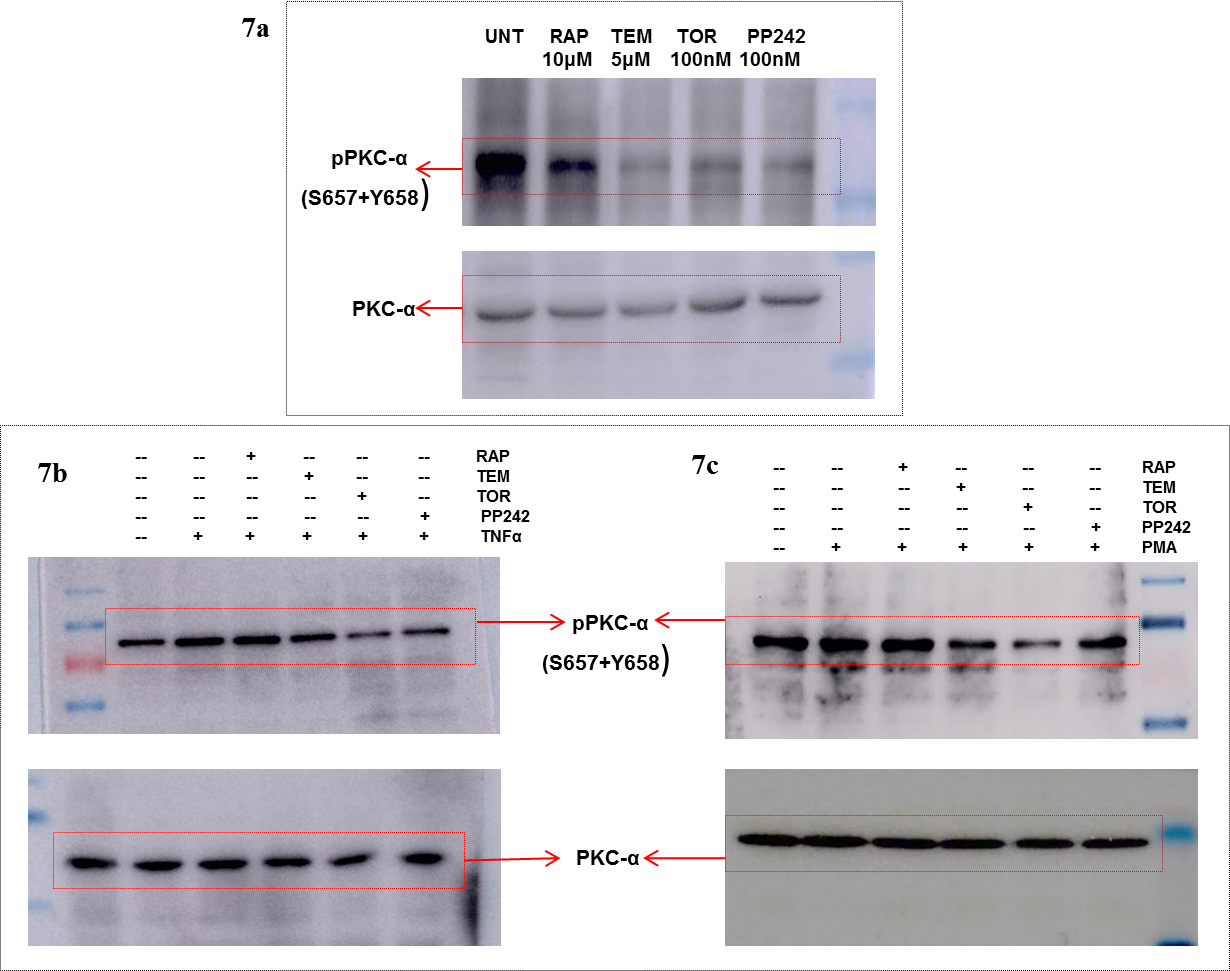


**Supplementary Figure S13.** Full-length blots relative to the cropped images showed in the main Figure 7a, 7b and 7c.

**Supplementary Table 1: Primers used for quantitative RT-PCR**

| **MMP-2** | **F primer: 5’-ATG ACA GCT GCA CCA CTG AG -3’**  **R primer: 5’-ATT TGT TGC CCA GGA AAG TG -3’** |
| --- | --- |
| **MMP-9** | **F primer: 5’-TTG ACA GCG ACA AGA AGT GG -3’**  **R primer: 5’-GCC ATT CAC GTC GTC CTT AT -3’** |
| **TIMP-1** | **F primer: 5’- CCA AGT TCG TGG GGA CAC -3’**  **R primer: 5’- TGC AGT TTT CCA GCA ATG AG -3’** |
| **TNF-α** | **F primer: 5’- AGG CCC CAG TTT GAA TTC TT -3’**  **R primer: 5’- TCC TTC AGA CAC CCT CAA CC-3’** |
| **IL1 β** | **F primer:5’- CCC TAG GGA TTG AGT CCA CA-3’**  **R primer:5’- AAG GCG GCC AGG ATA TAA CT-3’** |
| **VEGF** | **F primer:5’- TGT ATG TGG GTG GGT GTG TC-3’**  **R primer:5’- GGT CCC TCT TGG AAT TGG AT-3’** |
| **NOS-2** | **F primer:5’- TCC CGT CAG TTG GTA GGT TC-3’**  **R primer:5’- ACA AGC CTA CCC CTC CAG AT-3’** |
| **Cathepsin -B** | **F primer:5’- AAC CAC AGG CTG GGA TGT AG-3’**  **R primer:5’- CAC TGA CTG GGG TGA CAA TG-3’** |
| **Pentraxin-3** | **F primer:5’- TGA AGA GCT TGT CCC ATT CC-3’**  **R primer:5’- TGC GAT TCT GTT TTG TGC TC-3’** |
| **GAPDH** | **F primer:5’-ATG GGT GGA ATC ATA TTG GAA C-3’**  **R primer:5’-GAA GGT CGG AGT CAA CGG ATT T-3’** |
